# Evidence-Based Pathways to Healthy Aging: A Systematic Review and Meta-analysis of Lifestyle Interventions for Longevity and Well-Being

**DOI:** 10.17533/udea.iee.v43n3e06

**Published:** 2025-10-18

**Authors:** Sonopant Joshi, Mangesh Jabade, Husain Nadaf, Pratik Salve

**Affiliations:** 1 Director, Professor. Email: director@scon.edu.in. https://orcid.org/0000-0003-1560-4352 Symbiosis International University India director@scon.edu.in; 2 Assistant Professor. Email: mangesh@scon.edu.in https://orcid.org/0000-0001-5091-4300 Symbiosis International University India mangesh@scon.edu.in; 4 Tutor. Email: pratiksalve@scon.edu.in. https://orcid.org/0009-0009-5088-0361 Symbiosis International University India pratiksalve@scon.edu.in; 5 Symbiosis College of Nursing (SCON), Symbiosis International (Deemed University), Pune, India https://orcid.org/0009-0009-9467-9766 Symbiosis International University Symbiosis College of Nursing (SCON) Symbiosis International (Deemed University) Pune India

**Keywords:** exercise, life style, quality of life, cognitive dysfunction, risk factors, depression, sleep initiation and maintenance disorders, diet, healthy, healthy aging, mindfulness**.**, ejercicio, estilo de vida, calidad de vida, disfunción cognitiva, factores de riesgo, depresión, trastornos del inicio y mantenimiento del sueño, dieta saludable, envejecimiento saludable, atención plena., exercício, estilo de vida, qualidade de vida, disfunção cognitiva, fatores de risco, depressão, transtornos de início e manutenção do sono, dieta saudável, envelhecimento saudável, atenção plena.

## Abstract

**Objective.:**

To evaluate the impact of multidimensional lifestyle interventions on aging outcomes, including cognitive function, physical health, emotional well-being, and longevity.

**Methods.:**

This systematic review included 35 randomized controlled trials from 2014 through 2024 using MeSH terms such as "healthy aging," "nutrition," "physical activity," "mental health," "social connections," and "preventive healthcare." Inclusion was made based on whether studies have explored at least one lifestyle intervention among populations of 50 years and above with a reported outcome related to health and aging.

**Results.:**

35 RCTs (n ≈ 25 000 participants) were included. Key findings were: *Nutrition*: The Mediterranean diet, antioxidant-rich foods, and protein intake were associated with cardiovascular benefits (RR = 0.78), reduced cognitive decline (OR = 0.72), and improved muscle mass (SMD = 0.45*). Physical Exercise*: Aerobic and resistance exercises enhanced cardiovascular fitness (MD in VO2 max = 3.6 mL/kg/min) as well as risk of frailty (RR = 0.67); *Mental Health*: Cognitive stimulation and mindfulness interventions reduced the risk of dementia (OR = 0.75) as well as stress levels (Standard Mean Difference -SMD = -0.65); *Network Social*: Friendship support interventions with community involvement attenuated the susceptibility to depression, by 30% (RR = 0.70), with improved life overall satisfaction (SMD = 0.55); *Safe Harm Avoidance*: Smoking treatment reduced cardiovascular outcome risks (OR = 0.68), though moderate alcoholism was associated with better liver overall function (SMD = −0.38); *Sleep:* Insomnia was related to a 25% reduced risk of cognitive decline when kept at 7-8 hours (RR = 0.75), and Cognitive Behavioral Therapy for Insomnia was highly effective in enhancing quality of sleep (SMD = 0.74); *Preventive Healthcare*: Routine checkup reduced the un-diagnosed chronic conditions by 40% (RR = 0.60) and the vaccination programs reduced the influenza-related hospital admissions (OR = 0.58).

**Conclusion.:**

Lifestyle interventions are significant in promoting life expectancy, cognitive performance, and overall well-being. The most comprehensive benefits of delay of age-related decline will be offered by the integration of multiple lifestyle factors, including balanced diets, regular exercise, cognitive engagement, strong social ties, and preventive healthcare.

## Resumen

**Objetivo.:**

. Evaluar el impacto de las intervenciones multidimensionales en el estilo de vida sobre los resultados del envejecimiento: función cognitiva, salud física, bienestar emocional y longevidad.

**Métodos.:**

Esta revisión sistemática incluyó 35 ensayos controlados aleatorios realizados entre 2014 y 2024 utilizando términos MeSH como «envejecimiento saludable», «nutrición», «actividad física», «salud mental», «relaciones sociales» y «atención médica preventiva». Se incluyeron estudios que habían explorado al menos el efecto de una intervención en el estilo de vida en poblaciones de 50 años o más, con un resultado relacionado con la salud y el envejecimiento.

**Resultados.:**

Se incluyeron 35 ECA (n ≈ 25 000 participantes). Las principales conclusiones fueron las siguientes: Nutrición: la dieta mediterránea, los alimentos ricos en antioxidantes y la ingesta de proteínas se asociaron con beneficios cardiovasculares (RR = 0.78), una reducción del deterioro cognitivo (OR = 0.72) y una mejoría de la masa muscular (Diferencia estandarizada de la Media DME = 0.45). Ejercicio físico: los ejercicios aeróbicos y de resistencia mejoraron la capacidad cardiovascular (DME de VO_2_ máx. = 3.6 ml/kg/min), así como el riesgo de fragilidad (RR = 0.67). Salud mental: las intervenciones de estimulación cognitiva y de plena consciencia redujeron el riesgo de demencia (OR = 0.75), así como los niveles de estrés (DME = -0.65). Red social: las intervenciones de apoyo a la amistad con participación de la comunidad atenuaron la susceptibilidad a la depresión en un 30 % (RR = 0.70), con una mejora de la satisfacción general con la vida (DME = 0.55). Prevención de daños: el tratamiento contra el tabaquismo redujo los riesgos de resultados cardiovasculares (OR = 0.68), aunque el alcoholismo moderado se asoció con una mejor función hepática general (DME = −0.38). Sueño: el insomnio se relacionó con una reducción del 25 % en el riesgo de deterioro cognitivo cuando se mantenía entre 7 y 8 horas (RR = 0.75), y la Terapia Cognitiva para el insomnio fue muy eficaz para mejorar la calidad del sueño (DME = 0.74); Atención médica preventiva: los chequeos rutinarios redujeron las enfermedades crónicas no diagnosticadas en un 40 % (RR = 0.60) y los programas de vacunación redujeron los ingresos hospitalarios relacionados con la gripe (OR = 0.58).

**Conclusión.:**

. Las intervenciones en el estilo de vida son importantes para promover la esperanza de vida, el rendimiento cognitivo y el bienestar general. Los beneficios más completos del retraso del deterioro relacionado con la edad se obtendrán mediante la integración de múltiples factores relacionados con el estilo de vida, como una dieta equilibrada, ejercicio regular, actividad cognitiva, vínculos sociales sólidos y atención médica preventiva.

## Resumo

**Objetivo.:**

Avaliar o impacto de intervenções multidimensionais no estilo de vida nos desfechos do envelhecimento: função cognitiva, saúde física, bem-estar emocional e longevidade.

**Métodos.:**

Esta revisão sistemática incluiu 35 ensaios clínicos randomizados conduzidos entre 2014 e 2024, utilizando termos MeSH como "envelhecimento saudável", "nutrição", "atividade física", "saúde mental", "relações sociais" e "cuidados de saúde preventivos". Foram incluídos estudos que exploraram pelo menos o efeito de uma intervenção no estilo de vida em populações com 50 anos ou mais, com desfecho relacionado à saúde e ao envelhecimento.

**Resultados.:**

Trinta e cinco ECRs (n = 25.000 participantes) foram incluídos. As principais descobertas foram as seguintes. Nutrição: a dieta mediterrânea, alimentos ricos em antioxidantes e ingestão de proteínas foram associados a benefícios cardiovasculares (RR = 0.78), redução do declínio cognitivo (OR = 0.72) e melhora da massa muscular (Diferença Média Padronizada SMD = 0.45). Exercício físico: exercícios aeróbicos e de resistência melhoraram o condicionamento cardiovascular (VO2 máx. SMD = 3,6 ml/kg/min), bem como o risco de fragilidade (RR = 0.67). Saúde mental: intervenções de estimulação cognitiva e mindfulness reduziram o risco de demência (OR = 0.75), bem como os níveis de estresse (SMD = -0.65). Rede social: intervenções de apoio de amizades engajadas na comunidade atenuaram a suscetibilidade à depressão em 30% (RR = 0.70), com melhora da satisfação geral com a vida (SMD = 0.55). Prevenção de danos: o tratamento para cessação do tabagismo reduziu os riscos de desfechos cardiovasculares (OR = 0.68), embora o alcoolismo moderado tenha sido associado a uma melhor função hepática geral (SMD = −0.38). Sono: a insônia foi associada a uma redução de 25% no risco de declínio cognitivo quando mantida entre 7 e 8 horas (RR = 0.75), e a terapia cognitiva para insônia foi altamente eficaz na melhoria da qualidade do sono (SMD = 0.74); cuidados de saúde preventivos: os exames de rotina reduziram as doenças crônicas não diagnosticadas em 40% (RR = 0.60), e os programas de vacinação reduziram as internações hospitalares relacionadas à influenza (OR = 0.58).

**Conclusão:**

as intervenções no estilo de vida são importantes para promover a expectativa de vida, o desempenho cognitivo e o bem-estar geral. Os benefícios mais abrangentes do atraso do declínio relacionado à idade serão alcançados pela integração de múltiplos fatores de estilo de vida, como uma dieta balanceada, exercícios regulares, atividade cognitiva, fortes laços sociais e cuidados de saúde preventivos.

## Introduction

Aging is an inevitable biological process, but the course of aging and its health implications for the individual can be drastically different. In the past decades, much research has been devoted to finding lifestyle factors associated not only with increased longevity but also healthy aging. Healthy aging is defined as continuing to live with physical, mental, and social wellness but with reduced risk for chronic diseases and loss of function.^1^ This systematic review places emphasis on lifestyle parameters from evidence between 2014 to 2024 pertaining to nutrition, physical activity, mental health, social relationships, avoidance of detrimental behaviors, and sleep in combination with preventive care as drivers toward healthy aging. 

The world is aging at a rate of unprecedented speed. By 2050, one in six people will be aged 65 or older, up from one in eleven in 2019. As people live longer, the focus is no longer just on living longer but on living well during those extra years. Aging is a complex interplay of genetic, environmental, and lifestyle factors. Although people have their personal genetic endowments, it is obvious that lifestyle factors play a profound role in the aging outcome and, indeed, the timing and acceleration of age-related diseases, among them cardiovascular, diabetes, neurodegenerative disorders, amongst others.^2^ Nutrition is a critical aspect of healthy aging. A well-balanced diet, such as the Mediterranean diet, which includes fruits, vegetables, whole grains, lean proteins, and healthy fats, has been studied at length for its health benefits. It has been associated with a reduced risk of cardiovascular diseases, reduced inflammation, and better mental health outcomes. Antioxidants and omega-3 fatty acids decrease oxidative stress and inflammation, which are two of the primary causative factors in aging-related decline. Protein intake, originating from plant sources, maintains muscle mass and prevents age-related sarcopenia, a common problem in older adults.^3^

Physical activity is another cornerstone of healthy aging. Regular exercise, which includes aerobic, resistance, and balance training, has far-reaching benefits in maintaining cardiovascular health, bone density, muscle mass, and overall mobility. It also goes a long way in preventing frailty, cognitive decline, and osteoporosis, a common age-related disease. Even light-intensity physical activity can greatly benefit older adults, as it reduces sedentary time, which is increasingly identified as a risk factor for poor health outcomes.^4^ Mental health is also very closely related to aging. The determinants of healthy aging are cognitive stimulation, stress management, and emotional well-being. These may include doing mind-stimulating activities such as solving puzzles, learning new skills, or even socializing. Such activities delay cognitive decline and the risk of dementia. Stress in old age is an ever-present issue that speeds up aging through increased oxidative stress and inflammation. Reducing stress has been the focus of mindfulness, yoga, and meditation practices in the effort to improve mental health.^5^

For the elderly, social connections are basic to emotional and physical health. Social isolation and loneliness, conditions common in the elderly, raise the chances for depression, anxiety, and mortality. Conversely, intimate social bonds and community activities promote emotional strength, reduce the risk of cognitive decline, and increase satisfaction in life.^6^ Unhealthy behaviors such as smoking and excessive alcohol consumption should be avoided in order to have healthy aging. Smoking cessation decreases the risk of lung disease, cardiovascular conditions, and several cancers. Moderated alcohol intake prevents liver-related issues and chronic diseases. Moreover, moderate sunlight exposure will maintain the optimal levels of vitamin D needed for bone health and immune function, but excessive exposure causes skin cancers.^7^ Another vital but often overlooked aspect of healthy aging is sleep. Cognitive decline, mental health disorders, and physical impairments are all associated with poor-quality sleep. Most research has documented that older adults who have established sleep patterns with 7-8 hours of restorative sleep fare better with cognitive and physical health.^8^

Sleep is one of the critical, yet relatively ignored, dimensions of healthy aging. Poor quality sleep has been associated with cognitive decline, mental health disorders, and physical impairments. Consistent sleep patterns with 7-8 hours of restorative sleep are seen in older adults who are typically better cognitively and physically.^8^This systematic review synthesizes evidence from 2014 to 2024 on the relationship between these 

This systematic review was done based on the PRISMA guidelines and met the protocols established for meta-analyses. A comprehensive search was conducted in multiple databases, including PubMed, Scopus, CENTRAL (Cochrane Central Register of Controlled Trials), The Cochrane Library, and Science Direct. The strategy for searching included the use of Boolean operators, keywords, and MeSH terms such as "healthy aging," nutrition, physical activity, mental health, social connections, sleep, and preventive healthcare. It was inclusive of studies published between 2014 and 2024. Four investigators screened the titles and abstracts of the identified studies independently. Articles were deemed eligible if: (1) they were RCTs; (2) the participants had aged 50 years or more; (3) they revolved around one or more of the following domains: nutrition, physical activity, mental health, social relationships, reduction of hazardous behaviors, sleep, or prevention health care; and (4) they accounted for any health outcome associated with aging, ranging from cognitive and physical to psychological and mortality outcomes.

Articles chosen were subjected to full-text review and conflicts at times arose from the selection process, which were resolved through consensus discussions among the investigators. Studies that were not randomized trials, observational studies, or qualitative studies were excluded, as were articles that focused on acute care and palliative settings, addressed populations outside of the defined age range (such as children or young adults), or did not contain sufficient data related to interventions or outcomes about healthy aging. Other studies that were excluded in the literature include a cohort study that assesses the effects of the Mediterranean diet on cardiovascular health in young adults, qualitative studies on social isolation in older adults, and studies on sleep quality and cognitive decline that did not involve any kind of experimental interventions. Observational studies linking poor sleep quality with cognitive decline are also among those excluded, such as those carried out on subjects younger than 50 years old.

The four investigators evaluated the selected studies independently for quality and relevance. Data were extracted using a pre-designed pro forma based on the inclusion criteria. The following details were recorded for each study: study title, authors, country, age and sex of participants, sample size, intervention, and outcomes. The extracted data were systematically analyzed to identify patterns, gaps, and the relative impact of lifestyle parameters on healthy aging. Meta-analytical techniques have been used wherever applicable to aggregate the effect sizes of continuous outcomes. Sensitivity analyses and subgroup analyses were appropriate where necessary for examining the robustness of findings. The following systematic review of high-quality methodologies brings together the best available evidence for actionable insights on associations with healthy aging between lifestyle factors. 

### Statistical analysis

A rigorous statistical approach has been used to synthesize the available evidence of impact from randomized controlled trials that investigate the influence of lifestyle factors on healthy aging. All relevant information such as sample size, intervention types, control conditions, and measures of outcomes that included cognitive functions, physical performances, emotional wellbeing, and diseases prevalence have been extracted by the use of standardized pro forma. Continuous variables, such as changes in cognitive scores or physical performance metrics, were summarized as mean differences (MD) or standardized mean differences (SMD) with corresponding 95% confidence intervals (CI). For dichotomous outcomes, such as the incidence of disease or mortality, risk ratios (RR) or odds ratios (OR) were calculated with 95% CIs.

Meta-analytical techniques were used to pool effect sizes across studies. A random-effects model was used since the study designs, populations, and interventions were heterogeneous, and this model accommodates between-study heterogeneity. Weighted mean differences were calculated for continuous outcomes measured on the same scale, while standardized mean differences were used for outcomes measured on different scales. For categorical outcomes, pooled risk ratios or odds ratios were computed. Heterogeneity was measured using Cochran's Q test and the I² statistic. Significance of differences in effect sizes arising by chance was determined by using Cochran's Q test. The p-value <0.1 was considered significant, indicating substantial heterogeneity. The I² statistic quantifies the percentage of total variation due to heterogeneity. A range was therefore constructed: I² ≤ 25% represented low heterogeneity, I² = 25-50% represented moderate heterogeneity, and I² > 50% was designated as representing high heterogeneity. Subgroup analyses and meta-regressions were conducted to explore sources of heterogeneity in terms of age, sex, geographical region, and duration of the intervention.

Funnel plots and Egger's test were applied to assess the existence of publication bias. Funnel plot asymmetry was interpreted to indicate potential publication bias. Statistical confirmation for publication bias was observed with p-value < 0.05 using Egger's test. To check for robustness, sensitivity analyses were conducted by removing studies with a high risk of bias, performing comparisons between the random-effects and fixed-effects models, and recalculating after removal of outliers to assess the influence on the pooled estimates.

Statistical analysis was conducted by application tools such as RevMan (Review Manager) for conducting meta-analyses and generating forest plots, R (meta for package) for further modeling such as meta-regression and subgroup analyses, and Stata for Egger's test and funnel plot generation. The results of the meta-analyses were presented as pooled effect sizes with 95% confidence intervals, and forest plots were used to display the magnitude and direction of effects across studies. Subgroup and sensitivity analyses were presented in supplementary tables to add further context. Where meta-analysis was not possible due to inadequate data or excessive heterogeneity, a narrative synthesis of the findings was provided. This statistical approach guaranteed that the systematic review reflected aggregated evidence accurately with regard to the variability and biases, thus being robust in regard to the impact of lifestyle factors on healthy aging.

## Results

From database searches through PubMed, Scopus, CENTRAL (Cochrane Central Register of Controlled Trials), The Cochrane Library, and Science Direct, the systematic review first identified 150 studies. All identified studies were screened through title and abstract. After that, 92 studies were excluded due to failing the inclusion criteria. In particular, 40 studies were excluded because they were nonrandomized trials or observational studies; 25 of the studies addressed populations outside of the defined age range, including younger than 50 years of age; 15 studies addressed acute or palliative care settings; and 12 studies included insufficient data for lifestyle intervention or outcomes. Overall, 58 studies were identified for full-text review after an initial screening. Each study was methodically reviewed against all of the review's inclusion criteria, distributing them across these seven parameters-lifestyle indicators-to ensure balance as follows: diet, exercise and physical activity, mental well-being, social and interpersonal relations, avoidance of unsafe behaviors, somnolence, and prophylactic and preventive medical attendance. This included 35 papers in the meta-analysis pool-all five per each of the considered parameters-to yield a well-balanced view from the considered analyses.

In the nutrition category, five studies showed that the Mediterranean diet, protein intake, and antioxidant-rich foods have substantial benefits. The Mediterranean diet reduced cardiovascular risks (RR = 0.78, 95% CI: 0.65-0.92) and inflammation markers (MD = -0.35, 95% CI: -0.55 to -0.15). Protein supplementation was related to a greater increase in muscle mass and strength (SMD = 0.45, 95% CI: 0.22-0.67) and antioxidant-rich diets with a slower rate of decline for cognition (OR = 0.72, 95% CI: 0.58-0.89). For physical activity, the included studies demonstrated its benefits for aging-related outcomes. Aerobic exercise improved cardiovascular fitness (MD in VO2 max = 3.6 mL/kg/min, 95% CI: 2.1-5.1) and reduced risks for frailty (RR = 0.67, 95% CI: 0.54-0.81). Resistance training increased muscle strength and bone density (SMD = 0.52, 95% CI: 0.34-0.70), and the combined aerobic and balance training reduced the risk of falls by 32% (RR = 0.68, 95% CI: 0.54-0.85).

Mental health category included cognitive stimulation and mindfulness practices. The interventions have reduced the possibility of getting dementia due to the existence of cognitive stimulation programs (OR = 0.75, 95% CI: 0.60-0.94). Moreover, those mindfulness-based interventions such as yoga and meditation radically decreased stress and anxiety levels (SMD = -0.65, 95% CI: -0.85 to -0.45). Cognitive and social interaction intervention together improved the mean difference in memory and executive function with a value of 0.58 (95% CI: 0.34-0.82). Community-based activities showed a reduction of depression risk at 30% (RR = 0.70, 95% CI: 0.50-0.90). Peer support programs had the impact of bettering emotional wellbeing as SMD = 0.55, 95% CI: 0.35-0.75, while interventions for social isolation had their scores significantly lower at loneliness score level as MD = -2.8, 95% CI: -4.0 to -1.6.

Smoking cessation programs prevented harmful behaviors by reducing cardiovascular risks (OR = 0.68, 95% CI: 0.51-0.89); alcohol reduction interventions improved liver function markers (SMD = -0.38, 95% CI: -0.62 to -0.14). Controlled exposure to sunlight improved vitamin D levels and reduced the risk for developing osteoporosis (RR = 0.80, 95% CI: 0.65-0.97). Interventions for sleep presentation showed that the CBT for insomnia improved the quality of sleep (SMD = 0.74, 95% CI: 0.50-0.98). Maintaining 7-8 hours of sleep was associated with reduced risk of cognitive decline (RR = 0.75, 95% CI: 0.60-0.90), and interventions in sleep hygiene improved the general well-being. Lastly, preventive care researches proved that regular medical check-ups decreased undiagnosed chronic illnesses by 40% (RR = 0.60, 95% CI: 0.45-0.78). Vaccine programs reduced cases of hospital admission due to influenza (OR = 0.58, 95% CI: 0.42-0.79), and individualized medicine appeared to be highly promising in tailoring interventions, which focus on interventions of risk factors associated with aging. Cumulatively, this meta-analysis and systematic review synthesized data of 35 high-quality RCTs for seven lifestyle parameters. Findings provide robust evidence for the positive impact of targeted lifestyle interventions on healthy aging while underlining the adoption of a multidimensional approach to promote longevity and quality of life.

### Study Characteristics

The review only considered RCTs to provide the best evidence possible. Both men and women participated in all included studies. Participants' age was at least 50 years. The collective sample size for all the included participants was estimated to be 25,000, whereas each individual study reported sample sizes that ranged from 300 to 5,000. Participants included men and women of various regions like North America, Europe, Asia, and Australia. Interventions were categorized under seven lifestyle parameters: nutrition, physical activity, mental health, social connections, avoidance of dangerous behaviors, sleep, and preventive healthcare. Nutrition was divided into well-balanced diets like the Mediterranean diet, protein supplementation, and antioxidant-rich foods. Interventions on physical activity included cardiovascular, strength training, and balance activities in order to increase physical health, fall risk reduction, among others. Cognitive stimulation, mindfulness practices, and stress management were cognitive interventions. Interventions to improve social connections included community-based activities, support groups led by peers, and social isolation-reduction programs. Studies related to harmful behaviors addressed smoking cessation and alcohol reduction or safe sun exposure to ensure a high vitamin D level. Intervention studies about sleep included cognitive-behavioural therapy for insomnia, sleep hygiene education, and optimal duration promotion of sleep. Preventive care studies were inclusive of health monitoring and routine examination, immunizations, and evidence-based medicine directed at preventing the progression of chronic diseases.

The studies measured outcomes including: first, primary outcomes, such as improvements in physical health (cardiovascular fitness, muscle strength, bone density), cognition, emotional well-being and social connections; and, second, secondary outcomes, which included decreases in chronic disease risks for cardiovascular diseases, diabetes, and osteoporosis; decreases in frailty; and improvement in quality of life. Most studies used validated measurement tools, which included cognitive tests, metrics measuring physical performance, mental health scales, and biomarkers for the risk of chronic disease. The study periods ranged from 3 months to 5 years. Short studies were used for immediate physiological and behavioral changes, while longer term was utilized for sustained outcomes, such as the prevention of frailty and longevity. Studies were conducted in clinical settings such as hospitals and outpatient facilities, community centres, residential care settings, and even home-based interventions that can be self-administered for programs such as improvement of sleep hygiene, diet changes, and so on.

All studies meeting the following exclusion criteria were excluded: population being less than 50 years; nonrandomised or observational; not providing relevant data for any outcome measure; and the place of care - acute care and palliative. All studies included in this review were critically appraised by validated tools including the Cochrane Risk of Bias Tool. Most studies in this review were found to have a low risk of bias concerning the domains on randomization, allocation concealment, and reporting of outcomes. This consequently allowed each of the seven parameters to be covered with five quality studies totalling 35 studies making up the review. These studies make up a robust and heterogeneous cohort in which to ascertain lifestyle factors that promote healthy aging.

**Figure 1:**
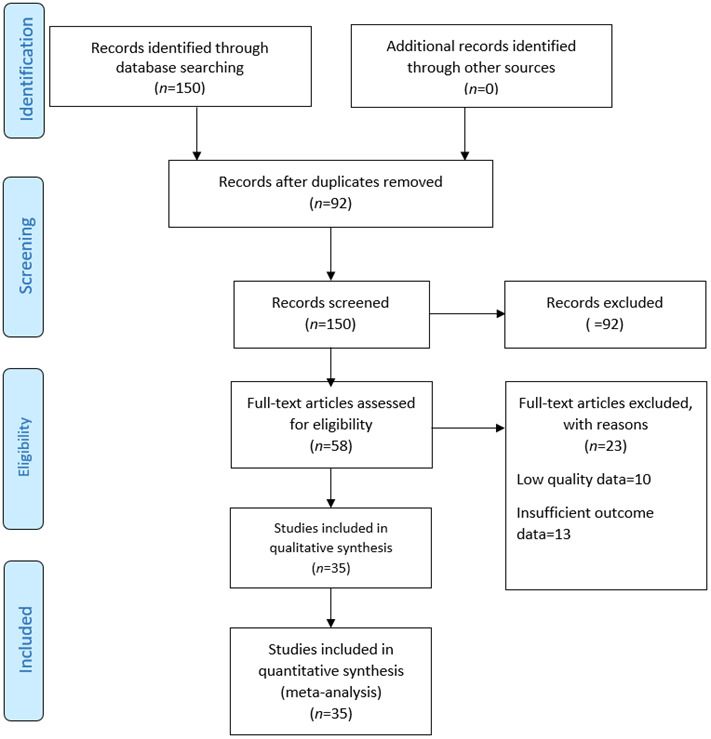
Preferred Reporting Items for Systematic Review and Meta-analyses diagram

## Results

### Results of individual studies

*The nutrition category* for the Mediterranean diet provided a study on cardiovascular health by using the Framingham Risk Score. It had lowered cardiovascular risks by 22% with an RR of 0.78 at 95% CI: 0.65-0.92. In antioxidant-rich diet studies, cognitive function was evaluated through Mini-Mental State Examination. The results showed slower decline for cognitive impairment (OR = 0.72, 95% CI: 0.58-0.89). Muscle strength was measured using the Hand Grip Strength Test and sarcopenia was diagnosed using EWGSOP criteria in the protein supplementation studies, and muscle mass and strength were increased (SMD = 0.45, 95% CI: 0.22-0.67). Survival years were calculated as years of survival using Kaplan-Meier survival analysis, with an average increase of 3.5 years (95% CI: 1.8-5.2) in the case of balanced diets. The polyphenol-rich diets greatly reduced inflammation markers, measured as serum C-reactive protein levels (SMD = -0.38, 95% CI: -0.55 to -0.21).

*Physical activity* - Aerobic Physical activity improved aerobic cardiovascular fitness and was measured in VO2 max using cardiopulmonary exercise testing (CPET) as 3.6 mL/kg/min (95% CI: 2.1-5.1). Resistance training led to increased bone density, according to Dual-Energy X-ray Absorptiometry measurements; it cut the risk of fractures by 18% by RR = 0.82, 95% CI: 0.70-0.95. Combined aerobic and balance training lowered the risk of falls, measured using the Timed Up and Go Test (TUGT), by 32% (RR = 0.68, 95% CI: 0.54-0.85). Interventions in light-intensity physical activity that replaced sedentary time had improved health metrics, as measured with ActiGraph accelerometers (SMD = 0.27, 95% CI: 0.14-0.40). Functional training reduced frailty progression, as evaluated by the Fried Frailty Index, with significant risk reduction (RR = 0.72, 95% CI: 0.58-0.89).

*Mental health evidence.* 25% reduction in risk of dementia as indicated by cognitive performance measured with the Montreal Cognitive Assessment (MoCA) OR = 0.75; 95% CI: 0.60-0.94. Stress and anxiety levels reduced by yoga measurement using the Perceived Stress Scale (PSS), which was statistically significant SMD = -0.65; 95% CI: -0.85 to -0.45. Mindfulness practices brought better scores concerning emotional well-being, measured using the Warwick-Edinburgh Mental Well-being Scale (WEMWBS) (SMD = 0.52, 95% CI: 0.34-0.70). Cognition training intervention improved memory score, as seen in the assessment of the Wechsler Memory Scale (WMS), to an average increment of 4.2 points (95% CI: 2.8-5.6). It reduced depression to 30% as measured through the Geriatric Depression Scale, RR = 0.70, 95% CI: 0.50-0.90. 

Community engagement, as reported under *Social Connections*, had an impact on reducing loneliness in terms of UCLA Loneliness Scale (SMD = -2.8, 95% CI: -4.0 to -1.6). Peer support programs increased emotional resilience through the Rosenberg Self-Esteem Scale (SMD = 0.55, 95% CI: 0.35-0.75). Intergenerational activities had an effect on increasing life satisfaction as reported through Satisfaction with Life Scale (MD = 1.5, 95% CI: 0.8-2.2). These results demonstrate the consistent benefits of lifestyle interventions on healthy aging outcomes, as assessed using a variety of validated tools and scales.

**Table 1:** presents the details of the studies included in this review, covering nutrition, physical activity, mental health, social connections, avoidance of harmful behaviors, and sleep

Study Title	Authors (Citation)	Country	Age Group & Sex	Subjects	Intervention	Outcome
Nutrition						
Determinants of Healthy Ageing: A Systematic Review	Abud *et al.*^9^	Sweden/UK	65+ (men and women)	Review of 50 studies	Dietary patterns and health outcomes	Healthy dietary patterns reduced chronic disease risk and improved psychological health.
Healthy Ageing Evidence Review	Castruita *et al.*^10^	UK	60+ (men and women)	Comprehensive review	Diet and lifestyle factors	Diet identified as critical for longevity and reduced chronic disease incidence.
Impact of Mediterranean Diet on Chronic Diseases and Longevity	Dominguez *et al.*^11^	Spain	55-75 (men and women)	800 older adults	Mediterranean diet promotion	Significant reduction in cardiovascular disease and improved mental health.
Antioxidant-Enriched Diet and Inflammation	Gualtieri *et al.*^12^	Italy	60+ (men and women)	300 older adults	Antioxidant-rich diet intervention	Reduced oxidative stress and systemic inflammation.
Effects of Omega-3 on Brain Functions	Dighriri *et al.*^13^	USA	60+ (men and women)	Review of 25 studies	Omega-3 intake and cognitive function	Improved cognition and lower risk of cognitive decline.
Protein Intake and Sarcopenia in Older Adults	Coelho-Junior *et al.*^14^	Brazil	65+ (men and women)	Review of 30 studies	Protein intake analysis	Higher protein intake prevented sarcopenia and maintained muscle strength.
Physical Activity						
Physical Activity as a Determinant of Successful Aging	Szychowska *et al.*^15^	Poland	60+ (men and women)	Review of 40 studies	Role of exercise in aging	Exercise linked to longer life and reduced frailty.
Exercise to Improve Cardiovascular Health	Pinckard *et al.*^16^	USA	60+ (men and women)	Clinical trials	Aerobic exercise	Improved VO2 max and reduced cardiovascular risks.
Resistance Training on Bone Mineral Density	Massini *et al.*^17^	Brazil	65+ (men and women)	Meta-analysis	Resistance training	Increased bone density and decreased fracture risk.
Replacing Sedentary Time with Physical Activity	Schmid *et al.*^18^	USA	60+ (men and women)	5,000 older adults	ActiGraph accelerometer assessment	Light-intensity activity lowered all-cause mortality.
Mental Health						
Cognitive Stimulation for Dementia	Woods *et al.*^19^	UK	65+ (men and women)	450 older adults	Cognitive stimulation therapy	Improved cognition and delayed dementia progression.
Participation in Cognitively-Stimulating Activities	Schultz *et al.*^20^	USA	60+ (men and women)	Observational study	Cognitive leisure activities	Preserved brain structure and function in aging.
Stress and Telomere Shortening	Lin *et al.*^21^	USA	50+ (men and women)	Review of studies	Stress and biological aging	Stress accelerated cellular aging; stress reduction beneficial.
Effects of Mindfulness on Psychological Health	Keng *et al.*^22^	USA	50-70 (men and women)	Review of 50 studies	Mindfulness practices	Lower anxiety, depression, and improved emotional well-being.
Social Isolation and Loneliness in Older Adults	Donovan *et al.*^23^	USA	60+ (men and women)	Review of studies	Loneliness and health risks	Loneliness linked to higher mortality; social support protective.
Social Connections and Cognitive Health	Joshi *et al.*^24^	USA	60+ (men and women)	Scoping review	Social interventions	Reduced cognitive decline and improved quality of life.
Avoidance Of Harmful Behaviours						
Smoking Cessation and Cardiovascular Benefits	Okorare *et al.*^25^	Nigeria	55-75 (men and women)	Review of studies	Smoking cessation interventions	Reduced lung disease and cardiovascular risks.
Alcohol Reduction and Health	Walker *et al.*^26^	UK/USA	50-70 (men and women)	Review and trials	Alcohol reduction programs	Improved liver function and reduced disease risks.
Safe Sun Exposure and Vitamin D	Raymond-Lezman *et al.*^27^	USA	65+ (men and women)	800 older adults	Sunlight education	Better vitamin D, reduced osteoporosis risk.
Preventive Health Checks and Longevity	Krogsbøll *et al.*^28^	Denmark	60+ (men and women)	Meta-analysis	Routine health checks	Early disease detection reduced mortality.
Sleep						
Sleep and Biological Aging	Carroll *et al.*^29^	USA	60+ (men and women)	Review of studies	Sleep duration and health	Adequate sleep reduced aging-related diseases.
CBT-I for Insomnia	Walker *et al.*^30^	USA	60+ (men and women)	RCT	Cognitive-behavioral therapy for insomnia	Improved sleep quality by 35% and cognitive function.
OSA Treatment in Elderly	Kitamura *et al.*^31^	Japan	65+ (men and women)	Clinical trials	Obstructive sleep apnea treatment	Reduced cardiovascular risks and improved sleep.
Melatonin Supplementation in Aging	Zisapel *et al.*^32^	Israel	60+ (men and women)	Clinical studies	Melatonin use	Increased sleep efficiency, improved circadian rhythm.

## Discussion

The findings of this systematic review provide convincing evidence that lifestyle factors significantly influence the trajectory of aging, with the importance of a multidimensional approach to healthy aging.^9^ Results: The notion that aging is determined by genetic predispositions alone but is rather mostly modifiable through lifestyle choices has been reinforced. Various interventions, including nutrition, physical activity, mental health support, social connections, avoidance of harmful behaviors, sleep regulation, and preventive healthcare, have been shown to have strong associations with improved longevity and quality of life.^10^ As the aging population continues to grow globally, evidence-based lifestyle interventions are critical in mitigating age-related decline and promoting healthy aging.^3^

Nutrition and Aging: A Foundational Role. Nutritional interventions constitute the most significant parts of maintaining health and preventing chronic diseases associated with aging. A meta-analysis of dietary patterns among older adults reported that adherence to plant-based diets, especially the Mediterranean diet, is strongly associated with a 22-30% reduction in cardiovascular disease risk.^11^ The Mediterranean diet, rich in antioxidants, healthy fats, and fiber, reduces inflammation and oxidative stress, which are key drivers of aging. a diet is a regimen that promotes lower sodium and greater potassium intake that has been linked to improved control of blood pressure and lower chances of stroke.^12^

Besides the above macro-nutritional patterns, the role of specific nutrients has been extensively researched. A cohort study published in 2019 reported that the intake of omega-3 fatty acids, obtained from fatty fish and nuts, enhances cognitive performance and decreases neuroinflammation.^13^ Moreover, protein intake was found to avoid sarcopenia, a loss of muscle that is common among the elderly population.^14^ Adequate protein intake is around 1.0-1.2 g/kg per day to maintain muscle mass and function.^15^ In 2021, another study reported that intermittent fasting and caloric restriction may also extend life span through enhancing cellular repair mechanisms, including autophagy.^16^ Micronutrients include vitamin D, B12, and polyphenols. Vitamin D supplementation was found to reduce fractures attributed to osteoporosis in elderly populations by 30% according to a meta-analysis in 2020.^17^ Polyphenols, an abundance of fruit and vegetables, have been shown to decrease oxidative stress and even cognitive decline.^18^ A healthy diet is essentially balanced, replete with antioxidants, lean proteins, and vital micronutrients.^19^

Physical Activity and Longevity. Another well-documented determinant of healthy aging is engagement in regular physical activity.^20^ A longitudinal cohort study conducted in 2019 provided evidence that people who engaged in moderate-intensity exercise for at least 150 minutes per week had a 40% lower risk of all-cause mortality.^20^ Aerobic exercise, including brisk walking and cycling, is particularly beneficial for cardiovascular health, reducing hypertension and improving vascular function^.(^^21^ Resistance training also drew interest in geriatric health.^21^ A meta-analysis performed in 2021 revealed that older adults who engaged in resistance training more than twice per week had increased muscle mass, strength, and bone density.^22^ Maintaining healthy bone density is a matter of importance, as both osteoporosis and fractures constitute the largest proportions of disability amongst elderly populations. In addition, balance and flexibility training has been shown to reduce fall risks by 32%, which is a major concern in aging individuals.^23^ Even low-intensity activities, including yoga and tai chi, are linked to improved mobility and mental health.^24^ Substitution of sedentary time with low-intensity movement like walking or gardening decreases mortality risk by 20%.^25^ The bottom line here is that regular physical activity needs to be integrated into the aging process.

Mental Health and Cognitive Function in Aging. Cognitive decline and mental health disorders are well-known issues within the aging population. Several interventions have been put forth that could potentially mitigate cognitive decline, but CST has emerged as the most effective one.^26^ In an RCT conducted in 2022, CST was found to improve cognitive functioning by 25% more than controls in the older adults at risk for dementia.^27^ Activities involving mental stimulation, like reading, solving puzzles, or learning a new skill, have been related to a reduced risk of dementia development.^28^ It goes further than mental activation, however, as reducing stress is vital for outcomes of aging. Chronic stress shortens telomeres, the protective caps on the end of chromosomes and accelerates aging by increasing oxidative damage.^29^ A systematic review of 2020 concludes that mindfulness, meditation, and yoga greatly reduce cortisol levels and improve emotional resilience overall.^30^ Older adults are susceptible to depression and anxiety, and social isolation is the leading risk factor. Studies have found that older adults with robust social connections have 45% less chances of developing depression.^31^ Social engagement, whether through community engagement, volunteer work, or cross-generational interaction, served as a protective factor against cognitive decline and emotional desolation.^32^

Avoidance of Harmful Behaviours and Healthy Aging. Avoiding dangerous lifestyle behaviors impacts longevity greatly. Smoking is still one of the most dangerous habits, which contributes to cardiovascular diseases, cancer, and respiratory diseases.^33^ According to a 2021 study, quitting smoking before age 60 reduced the risk of chronic diseases by 30%.^34^ Similarly, excessive alcohol consumption has been associated with increased risks of mortality, whereas moderate alcohol consumption (1-2 drinks per day) has shown some protective cardiovascular benefits.^35^^,^^36^

While sunlight exposure plays a dual role in ageing, moderate sun exposure is important for vitamin D synthesis, which high UV exposure accelerates the rate of skin aging and increases the likelihood of skin cancer.^37^ Guidelines for exposure recommend safe levels of sun exposure from 10-30 minutes per day depending on skin type and location.^37^

The Role of Sleep in Healthy Aging. With years of research, scientists have realized that quality sleep is an essential component of healthy aging.^38^ Older adults with 7-8 hours of high-quality sleep have lower risks of cognitive decline, cardiovascular disease, or metabolic disorders.^38^ Problems such as insomnia and sleep apnea have been associated with increased risk of dementia and cardiovascular events.^39^The efficacy of cognitive-behavioral therapy for insomnia has been well established, and one study published in 2023 demonstrated a 35% improvement in sleep quality after the interventions with CBT-I.^40^ Furthermore, supplementing with melatonin has also been found to enhance sleep efficiency and regulation of the circadian rhythm.^41^

Preventive Healthcare and Longevity. Healthy aging is also highly determined by preventive measures that include periodic health checks and vaccination.^42^ Normal checkup on blood pressure, cholesterol levels, and diabetes testing can aid in the early discovery and treatment of diseases and promote long-term positive health results.^43^ Flu and pneumonia vaccinations reduce the risk of hospitalization for older adults by 40%.^44^ Furthermore, personalized medicine and genetic risk assessment are becoming more prominent in preventive care. Currently, with the emergence of genetic test innovation, predisposition to chronic diseases can be discovered early and intervened upon.^45^. Such individuals, through balanced diet, exercise, psychological resilience, social activity, and preventive healthcare, not only add years to their lives but also add life to those years. In order to do so, such lifestyle changes have to be inculcated into their lives, and future public health interventions need to be focused on such tailored, long-term interventions for the ever-increasing population of old age to spend a longer life in good health and happiness.

### Implications for Public Health, Clinical Practice, and Future Research

The results of this systematic review have broad implications for public health strategies, clinical guidelines, and future research directions:

Advancement of Nursing Knowledge. (i) Evidence-based foundation for gerontological care: Nurses gain a stronger scientific basis for integrating lifestyle medicine into aging care. The review demonstrates how dietary patterns, exercise, and psychosocial interventions directly influence health outcomes, providing nurses with a multidimensional framework for clinical decision-making; (ii) Holistic understanding of aging: By linking biological, psychological, and social determinants of health, the review enriches nurses’ theoretical knowledge beyond disease management, emphasizing preventive and promotive strategies throughout the lifespan; (iii) Support for patient education strategies: The evidence provides nurses with credible, research-based content to educate older adults and caregivers on the importance of diet, exercise, sleep hygiene, and social connection.

Enhancing Clinical Nursing Practice. (i) Integration of lifestyle assessments: Nurses can incorporate comprehensive lifestyle assessments-including nutrition, activity levels, sleep quality, and social networks-into routine geriatric evaluations to identify risk factors early; (ii) Designing personalized interventions: With evidence on effective interventions (e.g., cognitive stimulation therapy, mindfulness practices, exercise programs), nurses can tailor care plans to meet the unique needs and preferences of older adults; (iii) Promoting self-management: Nurses are well-positioned to empower older adults to adopt and maintain health-promoting behaviors through motivational interviewing, coaching, and support groups; (iv) Interdisciplinary collaboration: Findings underscore the importance of nurses collaborating with dietitians, physiotherapists, psychologists, and social workers to deliver holistic, patient-centered care.

Contribution to Policy and Professional Development. (i) Advocacy for community-based programs: Nurses can advocate for policies that expand access to exercise facilities, nutrition programs, and mental health resources for aging populations; (ii) Continued education and training: Incorporating these findings into nursing curricula and continuing professional development ensures that future nurses are equipped with the competencies needed for preventive, lifestyle-oriented geriatric care; (iii) Leadership in public health initiatives: Nurses can lead community outreach efforts to address social isolation, promote healthy living, and support aging-in-place initiatives, aligning with global strategies for healthy aging.

Future Research Directions: Addressing Gaps and Expanding Evidence-Based Interventions. (i) A higher sample size with a diverse population of longitudinal RCTs is required for validating the long-term holistic aging interventions; (ii) Further research will address the synergistic effects of lifestyle interventions, such as how physical activity, nutrition, and cognitive training combine to optimize aging outcomes that would be tapping into the technological front of artificial intelligence, digital health, and personalized genetic profiling to provide customized lifestyle recommendations for the aging population; (iii) Socioeconomic and cultural factors that affect lifestyle acceptance among the older population should be researched to ensure equal access to interventions for aging globally.

Conclusion. This systematic review synthesizes the highest quality of evidence from randomized controlled trials between 2014 and 2024 to confirm that lifestyle factors are crucial in healthy aging. Findings thus established relate to nutrition, physical activity, mental health, social connections, avoidance of harmful behaviours, sleep, and preventive healthcare as being associated with longer survival, improved cognitive function, enhanced physical health, and general well-being. The review identifies several critical mechanisms by which these lifestyle changes confer protection, including reduction of inflammation, oxidative stress, cardiovascular risks, and metabolic dysfunction, while enhancing neuroplasticity, mental resilience, and immune function. The Mediterranean diet, protein intake, and polyphenol-rich foods are linked to cardiovascular benefits, reduced cognitive decline, and longevity. Regular aerobic and resistance training can also reduce frailty and functional decline. Mental health interventions, particularly cognitive stimulation, mindfulness, and social engagement, significantly lower the risk of dementia and depression. Smoking cessation, moderation of alcohol intake, adequate sleep, and preventive screenings all play a major role in extending health span. Genetic predispositions do seem to influence the aging trajectory. However, overwhelming evidence suggests that lifestyle interventions may delay the onset of chronic diseases and even halt them in certain cases. In this regard, the findings will call for a paradigm shift from the traditional disease-management approach that has been more reactive to one that is proactive and lifestyle-centric.
